# Control of Malaria Vector Mosquitoes by Insecticide-Treated Combinations of Window Screens and Eave Baffles

**DOI:** 10.3201/eid2305.160662

**Published:** 2017-05

**Authors:** Gerry F. Killeen, John P. Masalu, Dingani Chinula, Emmanouil A. Fotakis, Deogratius R. Kavishe, David Malone, Fredros Okumu

**Affiliations:** Ifakara Health Institute, Ifakara, Tanzania (G.F. Killeen, J.P. Masalu, D.R. Kavishe, F. Okumu);; Liverpool School of Tropical Medicine, Liverpool, UK (G.F. Killeen, D.R. Kavishe);; National Malaria Control Centre, Lusaka, Zambia (D. Chinula);; University of Crete Heraklion, Crete, Greece (E.A. Fotakis);; Innovative Vector Control Consortium, Liverpool (D. Malone);; University of the Witwatersrand, Johannesburg, South Africa (F.O. Okumu)

**Keywords:** malaria, Plasmodium spp., parasites, vector control, indoor residual spraying, insecticide resistance, lambda-cyhalothrin, pirimiphos-methyl, residual transmission, behavior, mosquitoes, Anopheles spp., Anopheles funestus, Anopheles arabiensis, window screens, eave baffles, entomology, vector-borne infections, Tanzania

## Abstract

We assessed window screens and eave baffles (WSEBs), which enable mosquitoes to enter but not exit houses, as an alternative to indoor residual spraying (IRS) for malaria vector control. WSEBs treated with water, the pyrethroid lambda-cyhalothrin, or the organophosphate pirimiphos-methyl, with and without a binding agent for increasing insecticide persistence on netting, were compared with IRS in experimental huts. Compared with IRS containing the same insecticide, WSEBs killed similar proportions of *Anopheles funestus* mosquitoes that were resistant to pyrethroids, carbamates and organochlorines and greater proportions of pyrethroid-resistant, early exiting *An. arabiensis* mosquitoes. WSEBs with pirimiphos-methyl killed greater proportions of both vectors than lambda-cyhalothrin or lambda-cyhalothrin plus pirimiphos-methyl and were equally efficacious when combined with binding agent. WSEBs required far less insecticide than IRS, and binding agents might enhance durability. WSEBs might enable affordable deployment of insecticide combinations to mitigate against physiologic insecticide resistance and improve control of behaviorally resistant, early exiting vectors.

Vector control with long-lasting insecticidal nets (LLINs) and indoor residual spraying (IRS) interventions account for 78% of the 663 million malaria cases and most of the 4 million deaths averted globally over recent years (*1*,*2*). LLINs and IRS can reduce malaria transmission by killing sufficient numbers of vector mosquitoes when they attack sleeping humans or rest indoors (*3*–*5*). However, as these approaches have been scaled up, physiologic resistance to insecticidal active ingredients has become increasingly common, threatening a “looming public health catastrophe” (*6*). Physiologic resistance to pyrethroids, the only class of insecticides suitable for use on LLINs, is now widespread and undermining vector control across Africa (*7*).

Only 4 directly lethal insecticide classes are recommended for control of adult malaria vectors with LLINs or IRS: pyrethroids (e.g., permethrin, deltamethrin, lambda-cyhalothrin); organochlorines (e.g., DDT); carbamates (e.g., bendiocarb, propoxur); and organophosphates (e.g., malathion, fenitrothrion, pirimiphos-methyl) (*8*). Mechanisms of cross-resistance against organochlorines and pyrethroids limit their utility for combined use in rotations, mosaics, or combinations (*7*,*8*). Organochlorines (especially DDT) and carbamates have a long history of use in agriculture and public health, and resistance to these classes is already emerging after only a few years of use in IRS at programmatic scales (*7*). However, these classes and organophosphates cannot be safely applied to LLINs at operationally effective doses (*8*), and are prohibitively expensive for routine IRS applications (*9*–*11*).

Year-round protection for the 40 million persons at risk of malaria in Tanzania, with IRS using the ideal recommended dose of the new capsule suspension formulation of the organophosphate pirimiphos-methyl, would cost US $157 million annually for insecticide procurement, exceeding the entire national malaria control budget of $114 million. Pirimiphos-methyl procurement for continuous IRS coverage of all at-risk populations would cost $3.3 billion annually across Africa and $12.5 billion worldwide, dwarfing the total global malaria control budget of $2.5 billion (*10*). As such expensive insecticides have become increasingly necessary because of pyrethroid resistance, IRS coverage has inevitably decreased (*9*–*11*) to only 3.4% globally (*12*). Although new insecticides are being developed for malaria vector control (*6*,*7*,*13*), these insecticides might also be similarly expensive. Unless new active ingredients are astutely delivered through rotations, mosaics, or combinations, they might not be any less prone to emergence of physiologic resistance (*6*–*8*).

Beyond physiologic resistance, effects of LLINs and IRS are also attenuated by the tendency of vectors to enter houses but then rapidly exit them, without resting on treated surfaces long enough to accumulate lethal doses of insecticide (*14*–*16*). Repeatedly entering and rapidly exiting several houses, until an unprotected human can be bitten, enables mosquitoes to mediate persistent residual malaria transmission by maximizing feeding opportunities while minimizing risks of exposure to LLINs and IRS when foraging indoors (*17*,*18*). Therefore, new insecticide delivery methods must target such evasive early exiting vectors (*14*,*16*), which might be described as behaviorally resilient (preexisting traits, typically with considerable phenotypic plasticity) or resistant (increasing frequency of selected heritable traits) (*17*,*19*). However, life history simulation analyses suggest such repeated visits to houses represent a vulnerability that can be exploited to great effect with improved methods for killing mosquitoes inside houses (*17*,*18*). Even for early exiting vectors, that often feed outdoors instead, most mosquitoes old enough to transmit malaria have previously entered >1 house, where they could be targeted with lethal insecticides or traps (*18*).

Personal protection provided by LLINs and IRS can be superseded and improved by physically mosquito-proofing houses with screened windows, ceilings, and closed eaves (*20*). However, most of the overall effects of LLINs and IRS on malaria transmission are achieved by killing mosquitoes en masse to protect entire communities, with more obvious contributions of personal or household protection being far less equitable and of lower magnitude (*4*). Household protection measures, such as spatial repellents or physical mosquito-proofing, which merely deter mosquitoes from entering houses and force them to seek blood meals elsewhere, might have less overall effect than measures that directly kill mosquitoes (*21*). In many settings with highly efficient vectors, elimination of malaria transmission will probably require lethal measures that suppress (*3*–*5*) or eliminate (*22*) mosquito populations, rather than merely deter them from entering houses (*21*). Therefore, new insecticide delivery methods are urgently needed to enable affordable deployment of multiple active ingredients and more effective targeting of early exiting mosquitoes (*6*,*8*,*13*).

We describe a simple housing modification with widely available netting materials that traps mosquitoes inside houses after they enter, and forces them into lethal contact with insecticides when they attempt to exit (Figure 1). Eave baffles have been used for decades (*23*) in standardized experimental hut designs for assessing LLINs and IRS (*24*,*25*). These baffles consist of netting panels slanting inwards and upwards from the upper end of the wall toward the roof, but leaving a small gap so that mosquitoes can freely enter the hut but cannot leave by the same route (Figure 1, panel A). Eave baffles have been used to target house-entering mosquitoes with fungal entomopathogens (*26*). In our study, baffles were combined with netting window screens and evaluated as a targeted delivery format for off-the-shelf formulations of commonly used chemical insecticides (Figure 1, panel B). This combination, called treated window screens and eave baffles (WSEBs), required far less insecticide than IRS. We assessed whether WSEBs could achieve control of physiologically resistant *Anopheles funestus* mosquitoes and early exiting *An. arabiensis* mosquitoes equivalent to that of IRS.

**Figure 1:**
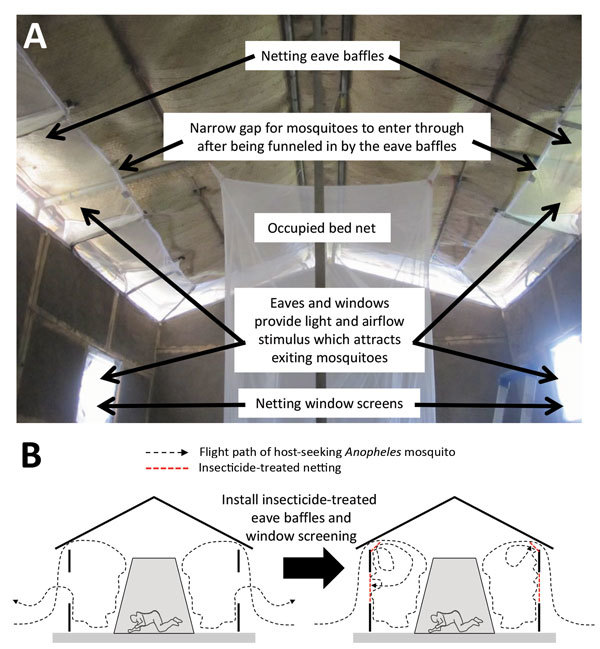
Design (A) and mechanism of action (B) of insecticide-treated window screens and eave baffles for control of malaria vector mosquitoes, Tanzania.

## Methods

All experiments were conducted in Lupiro village in the Kilombero Valley of rural southern Tanzania using commercially available IRS formulations of pyrethroids and organophosphates, which were combined with existing binding agent (BA) products for extending insecticide durability on LLINs. In this area of southern Tanzania, intense malaria transmission is mediated by 2 of the major malaria vectors in Africa. The first mosquito is *An. funestus*, which mediates rebounding (*14*) malaria transmission in this setting because it is physiologically resistant to pyrethroids, carbamates and organochlorines (*27*). The second mosquito is *An. arabiensis*, which mediates resilient residual transmission (*14*) because it is physiologically resistant to pyrethroids (*27*) and also exhibits early exiting behavior that renders it robust to indoor control with LLINs and IRS (*18*,*28*,*29*). All procedures were approved by the Institutional Review Board of the Ifakara Health Institute (IHI/IRB/34–2014) and the Medical Research Coordination Committee of the National Institute for Medical Research (NIMR/HQ/R.8a/Vol IX/1903).

We used 13 experimental huts of the Ifakara design (*24*,*29*,*30*) and standard methods (*31*) to assess effects of LLINs, IRS, and insecticide-treated WSEBs. Four of these huts were randomly selected, and their inner wall and roof surfaces were sprayed with 2 g/m^2^ of a capsule suspension formulation of pirimiphos-methyl (Actellic 300CS) by using standard programmatic application procedures (*32*). Another 4 randomly selected huts were sprayed with 30 mg/m^2^ of the pyrethroid lambda-cyhalothrin, which was also in a capsule suspension formulation (Icon 10CS). Both of these long-lasting, microencapsulated, insecticide formulations are manufactured by Syngenta AG (Basel, Switzerland) for IRS applications and are well characterized (*33*–*35*). The remaining 5 huts were sprayed only with water to serve as negative controls. After spraying, 2 mattresses and intact PermaNet LLINs (100-denier polyester multifilament mesh with 156 holes/inch^2^, surface-treated with 45−55 mg/m^2^ of deltamethrin in a resin foundation; Vestergaard, Lausanne, Switzerland) were installed in each hut.

Eave baffles are incorporated into experimental hut designs to ensure that mosquitoes can enter through approximately half of the eave gaps between the wall and the roof but are then all either retained in the hut or forced into interception traps fitted to the remaining exit points (*24*,*25*). In a conventional experimental hut study, those remaining exit points are windows and the remaining unbaffled half of eave gaps (*24*,*25*). However, the purpose of this study was to evaluate WSEBs as an insecticide delivery format. Therefore, all WSEB treatments, except for the negative control, included eave baffles fitted to all eave gaps, with and without exit traps, and identically treated screens fitted over all windows (Table; Figure 1). Treated WSEBs were fitted in front of exit traps, which were fitted immediately outside the hut (*24*), so that any mosquito attempting to exit through any eave gap or window would be forced into contact with these insecticidal netting barriers (Figure 1).

**Table:** Window screen and eave baffle treatments that were rotated through experimental huts with 3 IRS treatments for control of malaria vector mosquitoes, Tanzania*

Treatment no.	Description	Eaves baffled		Windows screened	Treatment of window screen and eave baffle netting		
		Entrances	Exits		LC, mg/m^2^	PM, g/m^2^	BA
1	Negative control: no trapping or insecticide	Yes	No	No	0	0	No
2	Partial negative control: trapping without insecticide	Yes	Yes	Yes	0	0	No
3	Partial negative control: trapping without insecticide	Yes	Yes	Yes	0	0	Yes
4	Trapping plus long-lasting LC and BA treatment	Yes	Yes	Yes	55	0	Yes
5	Trapping plus varying dose PM treatments	Yes	Yes	Yes	0	1	No
6		Yes	Yes	Yes	0	2	No
7		Yes	Yes	Yes	0	4	No
8	Trapping plus varying dose PM treatments with BA	Yes	Yes	Yes	0	1	Yes
9		Yes	Yes	Yes	0	2	Yes
10		Yes	Yes	Yes	0	4	Yes
11	Trapping plus varying dose PM treatments with BA and LC	Yes	Yes	Yes	55	1	Yes
12		Yes	Yes	Yes	55	2	Yes
13		Yes	Yes	Yes	55	4	Yes
*Indoor residual spraying treatments of experimental huts used lambda-cyhalothin (30 mg/m^2^ in 4 huts), pirimiphos-methyl (2 g/m^2^ in 4 huts), or a negative control (water diluent only: 5 huts), which was applied to all inner surfaces of walls and ceilings. All doses are per square meter of treated netting (window screening and eave baffles) or wall and ceiling surface (IRS), so that these doses can be directly compared in terms of lethality and cost per unit area treated. The 26-day schedule applied to complete 1 full replicate of evaluation for duplicates of these 13 treatments, by rotating them through all 13 IRS-treated experimental huts, is detailed in Technical Appendix 1. BA, binding agent; IRS, indoor residual spraying; LC, lambda-cyhalothin; PM, primiphos-methyl.							

The only treatment without screens over the windows or eave baffles over the half of the eave gaps with exit traps immediately outside were the negative control (Table). These controls had untreated eave baffles fitted only to the half of the eave spaces lacking exit traps, thus enabling mosquitoes to enter and exit. The 2 partial negative controls had screens fitted over the windows and baffles fitted to all eave gaps, regardless of whether they acted as entry or exit points for mosquitoes, but were not treated with any insecticides (Table). One partial negative control was treated with the noninsecticidal BA that Syngenta AG includes along with lambda-cyhalothrin (the same Icon 10CS formulation we used for IRS) in their Icon Maxx product to extend its active life on polyester netting (*36*).

The first insecticidal WSEB treatment (Table) was this same long-lasting Icon Maxx product, this time including both BA and lambda-cyhalothrin (*36*). Although the manufacturer-recommended dose of lambda-cyhalothrin on netting treated with the Icon Maxx product (55 mg/m^2^) is somewhat higher than that used for IRS (30 mg/m^2^), it is similar to that for deltamethrin on PermaNet LLINs used in this study (45-55 mg/m^2^). WSEBs treated with pirimiphos-methyl were assessed at 3 doses that were comparable with typical IRS application rates per square meter treated (Table). These 3 pirimiphos-methyl doses were also assessed as a co-treatment with BA to potentially extend insecticide life, with and without lambda-cyhalothrin as a complementary second insecticide from a different chemical class (Table). Lambda-cyhalothrin was chosen, despite being a pyrethroid to which both vector species in the study area are resistant (*27*), to assess the potential of such combinations to select for restored pyrethroid susceptibility (*37*). Conceptually, this approach relies on selectively reducing mortality rates for insects that are susceptible to its lethal mode of action and responsive to its irritant/repellent effects on mosquito behavior (*37*). The mathematical modeling study that motivated assessment of this combination assumed that these 2 pyrethroid susceptibility and responsiveness phenotypes, and presumably their underlying genotypes, are closely associated and therefore co-selected (*37*).

Although all exit traps on eaves and windows were made of Teflon-coated fiberglass mesh (*24*), all eave baffles and window screens were made of 100-denier polyester netting (A to Z Textile Mills, Arusha, Tanzania) of the kind typically used for bed nets. All WSEBs were treated by soaking in aqueous suspensions of the insecticides, BA, or both and then drying in the shade.

To execute the experimental design of this study, duplicate sets of the 13 detachable, movable WSEB treatments (Table) were rotated nightly through the 13 huts over two 26-day rounds of experimental replication (Technical Appendix 1) during December 5, 2015−February 1, 2016. Each night, 2 men (volunteers) slept under the 2 LLINs inside each hut from 7:00 pm to 7:00 am. These men then collected all mosquitoes inside the hut by using a Prokopak aspirator (John W. Hock Co., Gainesville, FL, USA) (*38*) and those inside the exit traps by using a mouth aspirator (*24*). Dead mosquitoes were then sorted taxonomically, classified by sex and abdominal status, and counted. Specimens collected alive were maintained in a field insectary for 24 h before separating live and dead specimens for sorting, classification, and counting. A random sample of 242 specimens from the *An. gambiae* complex was identified to sibling species by PCR (*39*).

Each pair of men remained assigned to a fixed experimental hut throughout the study so that variability associated with these volunteers and the huts could be analyzed as a single, consistent source of variance. After mosquito collection each morning, each pair of men was responsible only for installing the set of WSEBs assigned to their hut that evening and for removing that set from the hut it had been fitted to the previous night. All volunteers used a fresh pair of gloves each morning and were not allowed to handle any WSEBs other than those to be used in their hut that night. All WSEB sets were labeled and stored in labeled buckets during transfer between huts and the 13-day storage period of each 26-day replication cycle (Technical Appendix 1).

All field data were collected on hard copies of the adult field collection (ED1) and sample sorting (SS3) forms, recently described for informatically robust collection of entomologic data (*40*). To ensure rigid compliance with the experimental design, all attributes defined by it were prefilled into the forms (Technical Appendix 1). All statistical analysis was accomplished by using generalized linear mixed models with a binomial distribution and logit link function for the binary mosquito death outcome and fitted by using R version 3.2.1 (https://www.r-project.org/). WSEB treatments were included as categorical independent variables, and hut and night were included as random effects.

## Results

A total of 1,318 specimens from the *An. funestus* group and 5,842 from the *An. gambiae* complex were captured. Molecular identification confirmed continued absence of nominate *An. gambiae* mosquitoes in the study area (*22*). All of the 176 specimens that were successfully amplified (73% of the 242 specimens from this complex) were identified as *An. arabiensis* mosquitoes. All WESBs, other than the negative control, clearly retained mosquitoes within the huts, because this is where most (>90%) were collected, rather than in exit traps.

### Effects of WSEBs and IRS on *An. funestus* Mosquito Mortality Rates

**Figure 2:**
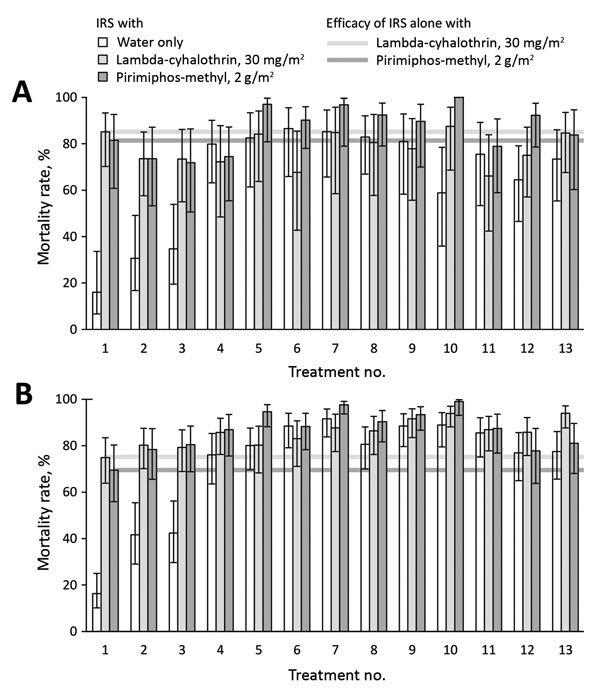
Effect of window screens and eave baffles treated with 13 combinations of insecticides and binding agents on malaria vector mosquito mortality rates inside experimental huts, Tanzania. A) *Anopheles funestus*. B) *An. arabiensis.* Huts were previously sprayed with 1 of 3 alternative indoor residual spraying regimens (Technical Appendix 1) and occupied by 2 volunteers sleeping under pyrethroid-treated, long-lasting insecticidal nets. IRS, indoor residual spraying. Error bars indicate 95 CIs. Estimated mean mortality rates and 95% CIs, as well as statistical contrasts between the most relevant treatment pairs, are indicated in Technical Appendix 2).

When used alone, most (8/10) WSEB treatments that included insecticides killed similarly high proportions of *An. funestus* mosquitoes as did IRS alone with the same insecticide formulations (Figure 2, panel A). Mortality rates for lambda-cyhalothrin plus BA-treated WSEBs alone were indistinguishable from those for lambda-cyhalothrin IRS (p = 0.363). The only exceptions among the 10 WSEB treatments were the highest pirimiphos-methyl dose plus BA and the intermediate pirimiphos-methyl dose plus lambda-cyhalothrin and BA.

Both of these WSEB treatments alone killed lower proportions of *An. funestus* mosquitoes than IRS with lambda-cyhalothrin alone; a similar but nonsignificant pattern was observed for comparisons of the same WSEB treatments alone with pirimiphos-methyl IRS alone (Figure 2; Technical Appendix 2). Nonetheless, mortality rates for pirimiphos-methyl–treated WSEBs alone were consistently high (Figure 2, panel A), regardless of treatment dose (p>0.156), and were statistically indistinguishable from pirimiphos-methyl IRS alone (p>0.713), even though the lowest WSEB dose per unit area treated was only half that for IRS. Although all combinations of pirimiphos-methyl–treated WSEBs with pirimiphos-methyl IRS resulted in higher mortality rates than pirimiphos-methyl IRS alone or pirimiphos-methyl–treated WSEBs alone, none of these differences were significant (p>0.080) because too few mosquitoes survived either treated WSEBs alone or IRS alone.

### Effects of WSEBs and IRS on *An. arabiensis* Mosquito Mortality Rates

Overall, insecticide-treated WSEBs either matched or were superior to IRS when used against *An. arabiensis* mosquitoes (Figure 2, panel B; Technical Appendix 2). WSEBs treated with lambda-cyhalothrin plus BA showed similar mortality rates as IRS with the same lambda-cyhalothrin formulation (p = 0.345). WSEBs treated with the lowest dose of pirimiphos-methyl showed similar mortality rates for *An. arabiensis* mosquitoes as IRS with twice as much pirimiphos-methyl per square meter treated (p = 0.419). However, increasing the pirimiphos-methyl treatment dose from 1 to 2 or 4 g/m^2^ increased the mortality rate for WSEBs (odds ratio [OR] 2.10, 95% CI 1.16–3.79, p = 0.0139; and 2.34, 95% CI 1.28–4.26, p = 0.0055, respectively), although there was no difference between intermediate and high doses (p = 0.758).

WSEBs with intermediate or high doses of pirimiphos-methyl killed more *An. arabiensis* mosquitoes (OR 5.9, 95% CI 1.4– 24.3, p = 0.0145; 10.8, 95% CI 1.6–74.8, p = 0.0157, respectively) than IRS, even though the intermediate pirimiphos-methyl dose was the same as for IRS per square meter treated. Supplementing pirimiphos-methyl–treated WSEBs with pirimiphos-methyl IRS increased *An. arabiensis* mosquito mortality rates for the lowest WSEB dose (OR 4.8, 95% CI 1.5–15.5, p = 0.0081), which was half that of IRS per unit area treated. However, supplementary pirimiphos-methyl IRS did not increase mortality rates when WSEBs were treated with the same dose as IRS (p = 0.748) or twice that dose (p = 0.429).

### Pirimiphos-Methyl Supplemented with BA and Lambda-Cyhalothrin as WSEB Co-treatments

Adding BA had no effect on the mortality rates for pirimiphos-methyl–treated WSEBs for *An. funestus* (p = 0.393) or *An. arabiensis* (p = 0.424) mosquitoes. Supplementing organophosphate pirimiphos-methyl plus BA treatment with the irritant pyrethroid lambda-cyhalothrin as a second active ingredient reduced *An. funestus* mosquito mortality rates for WSEBs (OR 0.64, 95% CI 0.46–0.89, p = 0.0076), presumably because the irritant properties of lambda-cyhalothrin reduced mosquito contact times with co-treated WSEBS, and therefore exposure to both insecticides. A similar but less dramatic, nonsignificant trend was observed for *An. arabiensis* mosquitoes (OR 0.88, 95% CI 0.73–1.06, p = 0.174).

## Discussion

Although WSEBs had higher efficacy than IRS against early exiting *An. arabiensis* mosquitoes, the 2 delivery formats had similar efficacy against *An. funestus* mosquitoes. Therefore, the most striking advantage of WSEBs is that they reduced the surface area treated per hut by >5-fold. Furthermore, co-application with existing BAs that already extend durability of pyrethroids on LLINs (*36*) for as much as 3 years (*41*) suggests new opportunities for reducing reapplication frequency by up to 6-fold, relative to IRS.

These WSEBs are an experimental prototype that were evaluated in the necessarily homogenous and controlled environment of experimental huts. This short-term efficacy study did not address key issues regarding potential effectiveness and cost-effectiveness of WSEBs under programmatic operational conditions. It is encouraging that a set of these WSEBs for these experimental huts, specifically designed to match the dimensions of local houses (*24*), required only 11 m^2^ of netting to manufacture, similar to a typical LLIN. However, this netting had to be carefully hand-tailored with hooks and Velcro to enable easy daily removal and reinstallation in experimental huts, at a manufacturing labor cost of $47 per set. More practical and affordable formats for operational use in a diversity of house designs must be developed and rigorously evaluated before WSEBs could be considered for routine, programmatic deployment by national programs.

Nevertheless, the potential of this approach merits consideration, even if only speculatively at this early stage. It takes almost an entire 833-mL bottle of the 0.3 g/mL pirimiphos-methyl formulation used here, costing ≈$24, to treat 1 typical rural house in Tanzania twice a year with IRS at the ideal recommended dose of 2 g/m^2^. In comparison, a house of equivalent size with WSEBs installed could be treated with the same insecticide at the same dose per square meter of treated netting for only $2.15. Although greater quantities of BA might be required than applied here (*42*), it could extend the life of pirimiphos-methyl on netting to the same extent as for lambda-cyhalothrin on LLINs that are approved for 3 years of use. If BA-treated WSEBs were similarly durable, they could provide up to 3 years of protection for only $0.72 per year in recurrent insecticide procurement costs. Because scale-up nationally in Tanzania would cost only $4.8 million for insecticide procurement, a combination of 3 similarly expensive complementary insecticides would be affordable to the national program at a cost of <$15 million annually. Corresponding global costs would be <$1.2 billion annually for such a triple combination.

Changing deployment format for existing IRS formulations could also eliminate the need to apply them in potentially hazardous aerosol form. Although handling insecticides is always associated with some risks, and protective clothing, eyewear, and a breathing apparatus might be required, WSEBs may be impregnated by simply dipping them in an aqueous suspension, similarly to bed nets. Therefore, WSEB deployment formats might enable national programs to develop and manage their vector control platforms more flexibly than when using IRS.

Although these insecticide cost estimates are entirely speculative, assume that BA will be equally efficacious for extending longevity of pirimiphos-methyl, and do not consider costs of netting installation or maintenance, they outline the potential economic benefits that could be accrued by optimizing WSEB deployment formats, netting materials, and treatment formulations. In addition, such reduced insecticide requirements might make rational resistance management (*8*) feasible and affordable with existing budgets and off-the-shelf insecticide products.

The observation that supplementing pirimiphos-methyl–treated WSEBs with the irritant pyrethroid lambda-cyhalothrin reduced mortality rates for *An. funestus* mosquitoes, which were strongly resistant to pyrethroids but not organophosphates (*27*), suggests that WSEBs could be used in an affordable format with which to field-test the theory that such combinations might select for restored pyrethroid susceptibility (*37*). The underlying assumption of this hypothesis is that physiologic susceptibility and behavioral responsiveness to pyrethroids are genetically linked, so that insecticide combinations, such as the LC-PM mixture used here, would selectively kill insects that are both resistant and non-responsive to pyrethroids.

The case for assuming that physiologic susceptibility and behavioral responsiveness are at least phenotypically associated has recently been strengthened by laboratory studies of *Culex quinquefasciatus* mosquitoes, which demonstrated that 4 pyrethroid-resistant field populations were all less responsive to the irritant properties of permethrin than a fully susceptible laboratory colony (*43*). These empirical studies (*43*) also suggest grounds for optimism regarding the recent theory that combining recently developed, low-technology emanators for airborne pyrethroid vapor (*44*,*45*) with complementary nonpyrethroid indoor control measures, such as IRS, WSEBs, or alternative technologies, such as eave tubes (*46*–*48*) and entry traps (*49*), could coselect for evolutionarily stabilized restoration of physiologic susceptibility and behavioral responsiveness to pyrethroids generally (*50*).

Genetic linkage between physiologic susceptibility and behavioral responsiveness to pyrethroids remains to be demonstrated. Also, both mathematical models predicting restoration of these preferred traits (*37*,*50*), by definition, merely illustrate the plausibility of these hypotheses in mathematically explicit terms. Alternatively, selection for physiologic resistance to both insecticides might be exacerbated by reducing contact exposure to sublethal levels. Although potential benefits and risks of combining irritant pyrethroids with nonirritant insecticides from complementary classes remain to be satisfactorily assessed, our results suggest that WSEBs might be a potentially scalable delivery format with which to test these hypotheses empirically through large-scale field studies.

Technical Appendix 1 Twenty-six–day schedule applied to complete 1 full replicate of evaluation for duplicates of the 13 treatments of window screens and eave baffles by rotating them through all of the 13 presprayed experimental huts for study of control of malaria vector mosquitoes by insecticide-treated combinations of window screens and eave baffles, Tanzania.

Technical Appendix 2 Estimated mortality rates of both malaria vector species in houses with each of the 39 combinations of treatments for indoor residual spraying and window screens and eave baffles, as well all practically relevant statistical contrasts between them, Tanzania.
